# Greenway Cyclists’ Visual Perception and Landscape Imagery Assessment

**DOI:** 10.3389/fpsyg.2021.541469

**Published:** 2021-05-21

**Authors:** Hui He, Jiamin Li, Xiaowu Lin, Yanwei Yu

**Affiliations:** ^1^School of Architecture & Urban Planning, Huazhong University of Science and Technology, Wuhan, China; ^2^Hubei Engineering and Technology Research Center of Urbanization, Wuhan, China; ^3^Xiamen Urban Planning & Design Institute, Xiamen, China

**Keywords:** visual perception, landscape imagery, greenway design, cycling, assessment

## Abstract

Greenway is a kind of corridors in the city that takes natural elements as the main constituent foundation and connects open spaces with functions such as leisure and recreation. The assessment of the built greenway is a review of the past construction experiences, and it is also a supplement and improvement to the future greenway planning concept system, which has important academic and application value. This study will explore how greenway design factors influenced the local cyclists’ perception of the landscape using on-site questionnaire and photo rating method. The results indicated that greenways with continuous cycling paths, high security awareness, open landscapes, and rich human activities evoke positive perceptions. Among the visual elements, natural elements such as plants and sky are more favorable than artificial elements. The research results show that the formation of greenway cyclists’ landscape imagery is affected by visual perception elements, which suggests that special consideration should be given to the laws of cyclists’ mental perception when designing greenways.

## Introduction

Environmental behavior studies and environmental psychology consider the interrelationship between people (users) and the physical (built) environment (Gifford, 1987; Moore, 1987; Yulian and Zhengfan, 2001; McCormack et al., 2010). This allows spatial environment research to be combined with environmental factors and cognitive behavior mechanism exploration, conducive to providing user-friendly design suggestions for space optimization and promotion.

The concept of greenway originated in the 1970s (Charles, 1995). It refers to corridors of linear green open space with ecological significance, leisure and recreation functions, and even historical and cultural values. Since 1985, Chinese scholars have successively introduced foreign greenway concepts, values, and practices, and have begun to make suggestions for improvement of urban green space systems and ecological infrastructure based on the principles of landscape ecology (Bin, 1985; Jianshuang and Fei, 2010; Kairan, 2010). Since 2009, China’s Pearl River Delta area^➀^ has been constructing greenways, a trend that has since expanded into Beijing, Wuhan, Chengdu, Changsha, and other cities (Xinhua News Agency, 2019).

Previous studies of greenways focused mainly on ecological network systems (Flores et al., 1998; Jim and Chen, 2003). However, with more attention given to the health of urban residents, research on environmental factors and healthy behaviors has begun to increase, and the fitness and sports function of the greenway has received more attention (Fitzhugh Eugene et al., 2010; Tully Mark et al., 2013; Frank et al., 2019; Hongyun et al., 2019). Research focusing on greenways for cycling space and other sports activities needs to be urgently undertaken.

Current research into the greenway cycling environment mainly relies on the comprehensive analysis of cycling environment element assessment and subjective assessment of cyclists (Tsui-Yun and Yann-Jou, 2007). There are three shortcomings in the research on greenway cycling (Moore, 1987) insufficient attention to the difference between greenway cycling and other physical activities (i.e., jogging, running) (Gifford, 1987) insufficient attention to the difference between greenway cycling and other types of cycling; and (Yulian and Zhengfan, 2001) the impact of landscape elements on cycling. In particular, the quantitative analysis of environmental elements of perception assessment is insufficient (He et al., 2019; Liu Song and Wanchen, 2020). The small number of case studies that fully consider the cyclist’s behavior and assessment perception limits the assessment of the cycling environment^➁^.

China’s official policy^➂^ specifies the facility configuration requirements in greenway design but lacks a scientific assessment system for the landscape quality generated by these elements. In essence, the understanding of the relationship between the quality of landscape design with users’ perception and assessment is still insufficient. This study investigated the influential factors and patterns of a cyclist’s visual perception of the greenway landscape to enrich the design theory and construction guidelines of urban greenways.

## Core Concepts

### Visual Perception

Visual perception is a physiological function, which relies on visual senses and information processing organs (Rogers, 2011). Visual perception is the main source of information, including color, shape, depth, and dynamics. This information is processed and interpreted to develop a deeper understanding of the objective world (Gogel, 1962; Wei, 2006; Lengen, 2015). Visual perception is accompanied by subjective correction of information translation, a topic that psychology and brain science scholars are committed to research (Tayal, 1972; Pylyshyn, 1999).

In the landscape field, the study of visual perception fundamentally explores the essence of beauty (favorability, attractiveness, or preference). Visual esthetic quality entails the philosophical debate between Platonic and Kant-style beauty^➃^ (Terry and Wither, 2001; Wang et al., 2006), thus promoting the fusion of perception research on subjective judgment and objective characteristics (Lange and Legwaila, 2012). The study of perceptual laws (Shen, 2005) has advanced steadily with improvements in science and technology, as new technologies, such as ophthalmography and holographic projection, are applied to this research field.

Landscape assessment is inseparable from analysis of multi-sensory elements, especially the visual elements (Gan et al., 2014). In terms of visual perception, there are two research difficulties. The first is the identification of visual perception elements (Yanga et al., 2009). Not all types of visual information can be accurately identified. Advances in image recognition technology mean surveys use photo data for machine analysis instead of traditional manual identification (Lai et al., 2014; Gebru et al., 2017), which allows the current mainstream research to obtain basic information such as color, attribute, quantity, and scale. Jun analyzed the calculation method of the green view index and proposed that the distance between the observer and the green environment could affect the final value (Bin-yi, 2015). This means that future research will be more closely linked with psychology and brain science to obtain more reliable data (Coltheart, 2004). The other research difficulty is the quantification of visual perception results. Liu’s research explained the principle of subjective perception in the translation of objective environmental information of landscapes. He established a quantitative assessment standard and index system and proposed a technical method for measuring and simulating landscape openness and serene ranking (Liu et al., 2017). This work plays an important role in the further construction of a multi-level weight evaluation system. There is still a lack of scientific and quantitative models, and further research on neurophysiological technology and brain imaging technology is needed (Taira, 2017).

Cycling is a type of motion and greenway cyclists often cycle with a speed of 10–25 km/h, so that the greenway becomes a fast-moving landscape (Jian, 2017). This differs from the exploration of moving objects in previous research on visual perception (Sachsenweger, 1986; López-Moliner et al., 2004), as cyclists are the moving subjects. The relationship between the environment and the cyclists can also change with the cycling speed, viewing direction, and angle of sight (Liang et al., 2002; Santillán and Barraza, 2019).

### Landscape Imagery

The concept of landscape is the whole of its form and function, with the totality of subjective and objective interaction, and being perceived is one of its inherent attributes^➄^. Whether in eastern or western culture, the earliest meaning of landscape derives from visual esthetics. During the Renaissance, landscape gradually became an esthetic object from the background of human existence, and natural landscape gardens became one of the expressions of human artistic esthetics (Pedroli and Pinto-Correia, 2006; Stobbelaar and Pedroli, 2011). In 1935, Chinese silviculturist Chen Zhi’s *Introduction to Gardening* introduced the Japanese character “landscape” into China. Chen Zhi’s work described the basic meaning of traditional gardens and landscapes and introduced characteristics of visual esthetics (Zhi, 1935; Guang-si, 2006). Landscape imagery is a composite concept and an important influence in the conception and formal expression of landscape design, audience perceptual experience, and consciousness (Michael and Stephen, 1994).

Research on landscape imagery mostly focuses on the perception of physical landscape environment, reflecting a consensus regarding landscape consciousness (Nohl, 2001; Lei, 2014). Ding believes that the generalization and abstraction of specific landscape images can reflect the local cultural and historical characteristics and can be understood as a specific “landscape imagery unit” of the place (Shao-gang, 2011).

Landscape imagery assessment research methods commonly use social investigation and statistical analysis, such as importance-performance analysis (O’Leary and Lee, 2016) and semantic differential (SD) methods (Cao and Liang, 2004). These methods try to translate the viewers’ perception results into quantitative indicators through semantic descriptions.

Considering that perceptual evaluation is a kind of subjective evaluation, research will generally adopt public preference surveys to enhance the effectiveness of evaluation (Clay and Daniel, 2000; Kalivoda et al., 2014; Gao et al., 2019). Xu uses the SD method and scenic beauty estimation to explore the esthetic differences underpinning landscape assessment and proposed that landscape design should pay more attention to the diversity of user types, considering both functionality and esthetics (Wang et al., 2006; Da-wei, 2014; Vessel et al., 2018).

In the study presented here, the differences in the elements of the perception results of different observers will be summarized, some image structure models will be drawn, and the overall image space cognition results will be further explored (Chaolin and Guochen, 2001; Wei, 2006; Yuehao et al., 2019).

## Materials and Methods

### Study Area (Site)

The 102-km Eastlake Greenway, located in the city center of Wuhan Eastlake Ecological Tourism National-level Scenic Area^➅^, is the “ecological green heart” for locals.

All the loops of the greenway connect the five major areas of the overall scenic area where one can enjoy a variety of natural sceneries: mountains, lakes, forests, wetlands, islands, and fields (Wuhan Donghu Green Road Operation Management Co., Ltd., 2019). UN-Habitat described Eastlake Greenway as “China’s demonstration of improving urban public space.” (UNHABITAT, 2019) Contrasted with countryside greenway, it is a unique city greenway with entrances distributed over three districts in Wuhan for easy access for its citizens (Chao and Xuan, 2017). Our preliminary studies showed that more than 60% of users chose to cycle along the greenway (He et al., 2019).

**FIGURE 1:**
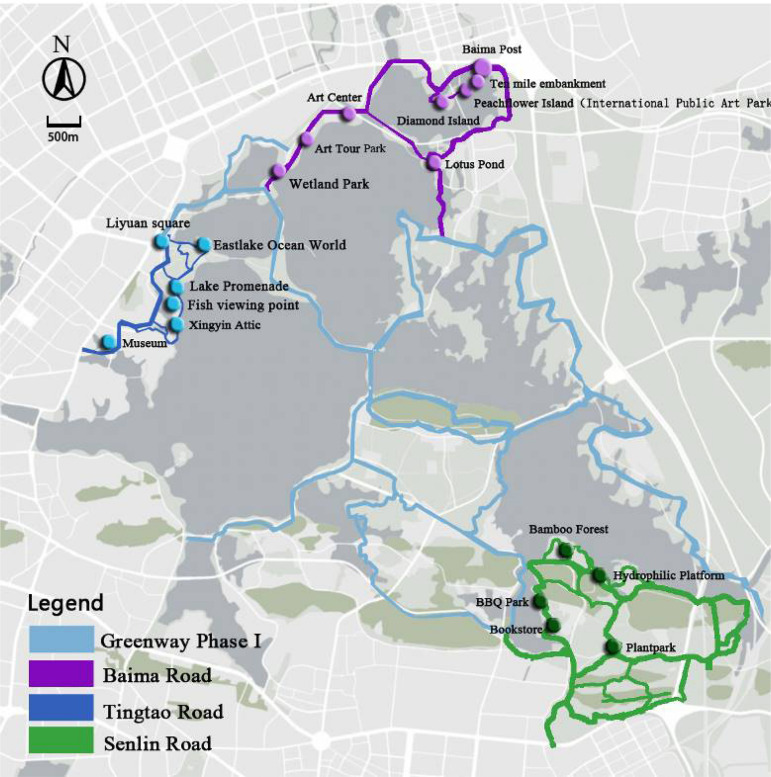
Eastlake Greenway Location and Construction (Nodes indicate locations of scenic spots).

The greenway is divided into two phases (Phase I finished in 2016, and Phase II in 2017) (Figure 1). The research object in Phase II consists of three parts: Baima Road, Tingtao Road, and Senlin Road (Wuhan Donghu Green Road Operation Management Co., Ltd., 2019)^➆^.

Baima Road is 14.7 km and mainly includes two islands and a lake-long embankment with miles of peach blossoms. This area was designed as a city waterfront ecological art zone and provides an important image of an urban portal because of its location. Tingtao Road is 9.02 km, and was designed as an urban garden, combining historical culture with natural scenery for public fitness and enjoyment. Senlin Road is 26.22 km and located in a natural forest park. It connects multiple botanical gardens and a series of outdoor activity areas.

Phase II is described as wilder and fun in that it is more closely connected with nature than Phase I. The visual landscape is more beautiful, and the cultural connotations are more abundant. In terms of the cycling experience, Phase II has more gentle trails, the natural ecology is plentiful, and there are a greater variety of route options for ecological exploration and reflection (Hongxia and Jingang, 2019; Xiao, 2019).

### Methods

Based on the research purpose of exploring the relationship between greenway cyclists’visual landscape elements and imagery perception assessment, this study mainly used comprehensive landscape analysis and assessment (Jiong-wei et al., 2010). The overall evaluation belongs to post-use evaluation (POE) and case empirical investigation. The main research methods can be introduced in three stages:

The first was to collect information through random questionnaire surveys, including cyclists’ basic socio-economic attributes, and greenway use characteristics – especially cycling activity characteristics during specific time periods (working days and non-working days) – for subsequent observation and comparison.

Second, for the study of visual landscape elements, this study mainly considered the impact of the environment on cycling activities. Thus, we did not focus on the ecological nature of the landscape, or the combination of vegetation, biodiversity, and accessibility studies. The compound effects of non-visual factors were not considered, as we rather tried to avoid the interference of these factors (choosing survey days with similar weather conditions). Therefore, in addition to recording the types and characteristics of the elements, the observation of visual elements itself contained the subjective assignment of the investigator, especially esthetic imagery perception (Kongjian, 1986). Past research typically used comprehensive qualitative and quantitative assessment (Genoveva, 1995; Gobster Paul, 1999; Akbar and Hale, 2003). Specifically, before the survey, the effect of the visual landscape was clarified using pictures with the upper and lower limits of the score, and scores were assigned based on this reference. In addition, with the help of image analysis technology and expert scoring methods, computer programs were used to analyze and calculate the visual elements of the on-site (node) photographs taken by the investigators on the field survey. At the same time, planning and design professionals scored the visual quality of these photographs as a reference for evaluation.

In our survey, visual elements were classified into natural elements and artificial elements (Table 1). Each element was scored and semantically described, including shape, color, attribute, scale, and intuitive feeling. Semantic statistical node description was used to determine the type of information that will receive more attention when scoring quality in the visual landscape.

**TABLE 1:** Visual landscape element evaluation table.

Node number:◻	Type	Score& description *Suggested description
Natural Elements	Mountain	*Height, slope
	Water	*Color, clarity, openness
	Plants (i.e., trees, shrubs, lawns, flowers)	*Richness, matching degree, sense of space
	Animals	*Type, psychological feeling
Artificial Elements	Facilities (Environmental, Rest, Guide facilities)	*Type, fashionable
	Vehicles	*Quantity
	Roads	*Width, material, color, straightness, slope
	Fence or block	*Function, form, psychological feeling
	Buildings (commercial, public service)	*Function, form, psychological feeling
	Landscape sketches (sculptures, water features, lights, flower stands, scenery walls, leaking windows)	*Type, fashionable
	Parking, roadblocks, telephone poles	*Type, fashionable
Pedestrians	Behaviors	*Quantity

Third, the SD method was mainly adopted for the evaluation of image perception (Junhua, 2004) and cognitive map technology (Lynch, 1960). Image perception described the subjective feelings of the cyclists in the photographs of the nodes with semantic word pairs, and assigned specific scores to obtain their quantitative perceptual evaluation. This is actually the establishment of a “transformation box” that converts cognitive feelings that are not easily expressed – including semantic adjectives, ordering of judgments, value orientation, and cultural associations – into relatively visible, computable scores (Tveit et al., 2006). A five-point assessment scale was used in this research, with scores of -2, -1, 0, 1, and 2, representing “very poor,” “poor,” “average,” “good,” and “very good,” respectively. The scoring was applied to 22 assessment items (Table 2), and the final score was divided into eight intervals (categories) from lowest to highest. A cognitive map was used with interviewees who were asked to mark the most memorable spatial points on the map with text information removed, and to try to describe and evaluate these anchor points^➇^, before finally superimposing these nodes on the space to form an imagery structure.

**TABLE 2:** Semantic assessment items and adjective pairs.

Assessment items	Semantic adjective pair	Score (−2∼2)	Assessment items	Semantic Adjective Pair	Score (−2∼2)
Sense of Space	Open∼Closed	◻◻◻◻	Naturalization	Natural∼Artificial	◻◻◻◻
Lightness	Bright∼Dark	◻◻◻◻	Vegetation Cover	High∼Low	◻◻◻◻
Layering	Layered∼Blurred	◻◻◻◻	Esthetic Sense	Beautiful∼Devoid of Beauty	◻◻◻◻
Rhythm	Strong Rhythm∼Weak Rhythm	◻◻◻◻	Coordination	Coordinated∼Disharmony	◻◻◻◻
Ambiguity	Atmosphere∼No Atmosphere	◻◻◻◻	Pleasure	Pleasant∼Unpleasant	◻◻◻◻
Color Richness	Colorful∼Monochrome	◻◻◻◻	Closeness	Close∼Alienated	◻◻◻◻
Continuity	Continuous∼Discontinuous	◻◻◻◻	Impression	Impressive∼Weak Impression	◻◻◻◻
Neatness	Accordant∼Messy	◻◻◻◻	Change	Variable∼Lack of Change	◻◻◻◻
Attractiveness	Attractive∼Unattractive	◻◻◻◻	Quietness	Quiet∼Noisy	◻◻◻◻
Vitality	Living∼Lifeless	◻◻◻◻	Dynamic	Dynamic∼Not Dynamic	◻◻◻◻
Freshness	Novelty∼Ordinary	◻◻◻◻	Security	Safe∼Unsafe	◻◻◻◻

*^∗^Score“-^∗^2, -^∗^2, 0, 1, 2” represent “very poor, poor, general, good, and very good”.*

Through the greenway cyclists’ visual landscape elements and imagery perception assessment, a spatial correspondence was established and used to explore whether there is a relationship between the two, and whether there are differences in the degree of influence of different types of visual landscape elements. This stage will simply make preliminary judgments using statistics (SPSS correlation analysis) without in-depth exploration.

### Data Collection

Data collection was mainly completed through field surveys that investigators chose to undertake on working and non-working days in May 2019^➈^.

**FIGURE 2:**
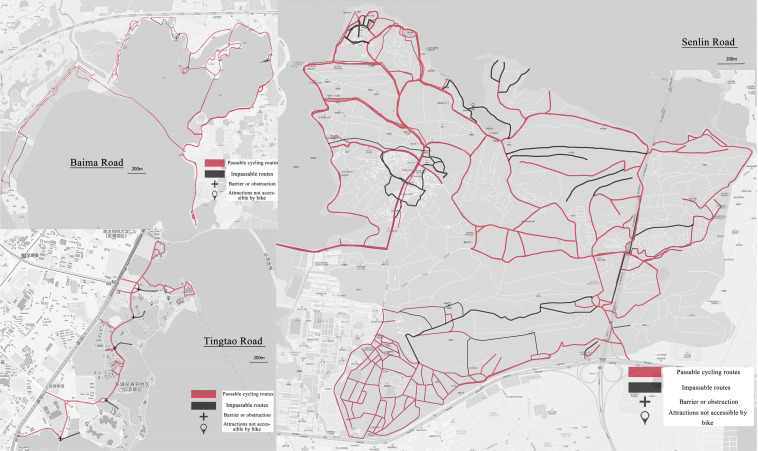
Baima Road, Tingtao Road and Senlin Road portable routes.

A total of 64 professional students were divided into two groups, a recording group and a questionnaire interview group. The recording group took 1289 photographs of 678 nodes and evaluated features from a cycling perspective^➉^. To ensure a sufficient number of pictures, the node photographs needed to contain records of at least every 100 meters. Therefore, it was found that there are differences in the continuity of the cycling routes (Figure 2). There are some routes in the three greenways likely to affect the subjective feelings of cyclists because of the slope, pruning, and impassable conditions such as road maintenance. Professional students also took 672 node pictures with higher visual quality based on their own judgment. The questionnaire interview group randomly distributed questionnaires and cognitive maps to greenway cyclists to obtain basic user information and use characteristics. They focused on recording the visual and semantic description of the nodes drawn by the cyclists. In the end, 237 valid questionnaires and 129 cognitive maps were obtained^⑪^.

## Results

### User Characteristics

User characteristic data comes from questionnaire interviews that included four main parts: basic information about cyclists, characteristics of greenway use, comprehensive scoring and semantic description selection, and cognitive maps of the three greenways.

#### Basic Information About Cyclists

Based on previous studies and our own observations, most cyclists were local citizens. Additional information, such as gender, was collected through the questionnaires (Table 3). Slight differences in gender, age, and work attributes may reflect variations in the public’s enthusiasm for the greenway space and the experiences it offers for all ages and interests.

**TABLE 3:** Basic information of cyclists.

Category	Weekend			Workday		
		
	Baima road	Tingtao road	Senlin road	Baima road	Tingtao road	Senlin road
**Gender**						
Female	44.12%	40.7%	40.3%	29.2%	33.3%	41.1%
Male	55.88%	59.3%	59.7%	70.8%	66.7%	58.9%
**Age**						
Under 18	0	3.7%	8.33%	4.17%	0	3.57%
18–40	73.5%	88.9%	75%	62.5%	87.5%	75%
40–60	20.58%	7.4%	16.67%	20.84%	12.5%	17.86%
Over 60	5.88%	0	0	12.5%	0	3.57%
**Occupation**						
Non-design related	94.1%	88.9%	84.7%	66.7%	83.3%	78.6%
Design related	5.9%	11.1%	15.3%	33.3%	16.7%	21.4%

The statistical results show the gender ratio for cycling on weekends is very similar, while more men cycle on workdays than women. The 18–40 age group comprises the main user group, and most people were non-professionals, which conforms to general survey expectations.

#### Characteristics of Greenway Use

Through the questionnaires, a simple understanding of the cyclists’ behaviors was obtained (Table 4). The scenic area service coverage is expansive, and the cyclists sampled were in a limited area. The main functional needs of the greenway specified by the cyclists were physical exercise and enjoyment of beautiful scenery. Due to differences in functional requirements, work schedule, transportation costs, and riding frequency were higher during the weekends when the time spent cycling for exercise was usually 1–2 h. The viewing route covered by these cyclists tended to be relatively long, and the speed was relatively fast. More leisurely cycling activities were influenced by the greenway nodes, rest facilities, interaction with the environment, and other behaviors interspersed throughout the leisure cycling excursions, when the ride time was relatively short.

**TABLE 4:** Characteristics of greenway use.

Category	Weekend			Workday		
		
	Baima road	Tingtao road	Senlin road	Baima road	Tingtao road	Senlin road
**Source**						
Less than 10 km	88.24%	70.4%	88.9%	87.5%	80.4%	75%
10–30 km	5.94%	25.9%	6.5%	12.5%	11.3%	3.57%
Over 30 km	5.82%	3.7%	4.6%	0	8.3%	21.43%
**Frequency**						
More than 3 times a week	23.53%	7.41%	16.67%	33.3%	29.17%	25%
About 1–3 times a week	20.59%	22.22%	20.83%	20.83%	4.17%	10.71%
About once a week	23.53%	22.22%	27.78%	8.33%	12.5%	17.86%
Less than once a week	2.94%	48.15%	34.72%	37.5%	54.2%	46.43%
**Goal(Multiple choice)**						
Exercise	76.47%	55.56%	66.7%	45.8%	37.5%	57.14%
View	67.65%	61.2%	48.6%	45.8%	41.7%	49.91%
Others	9.86%	23.21%	13.6%	12.67%	11.67%	11.79%
**Riding time**						
Within 30 min	3.88%	14.81%	12.5%	8.33%	33.3%	23.21%
30–60 min	23.53%	44.44%	39.11%	29.71%	37.5%	22.63%
60–120 min	46.12%	11.11%	26.65%	26.6%	12.6%	25.71%
More than 2 h	26.47%	29.63%	21.74%	35.36%	16.67%	28.45%

#### Comprehensive Scoring and Semantic Description Selection

Cyclists’ perception scores were determined over a relatively short time, so their recorded experience tended to be more general, resulting in a rough estimate of their overall perception of the landscape (Table 5). The results revealed some common elements of cyclists’ perceptions among the three roads: an appreciation of the greenway’s beautiful natural scenery and the good air quality for certain social leisure activities. Adjectives used to describe the cyclists’ experience of the landscape frequently included words such as “pleasure,” followed by “close” and “vibrant.” Cyclists generally believed that the first thing that attracted them during their cycling excursion was the presence of rich natural elements, followed by the designed plantings/gardens and waterfront areas. Most cyclists believed the greenway to be unique in its beauty, superior to typical parks and sports venues.

**TABLE 5:** Cyclist’s comprehensive scoring and semantic description selection.

Category	Weekend			Workday		
		
	Baima	Tingtao	Senlin	Baima	Tingtao	Senlin
	road	road	road	road	road	road
Average rating (Full score is 10)	7.86	7.62	7.95	7.75	7.71	7.81

Best service top 3	➀Natural scenery, ➁air quality, ➂social leisure	➀Natural scenery ➁air quality ➂social leisure	➀Natural scenery ➁air quality ➂social leisure	➀Natural scenery ➁air quality, ➂social leisure	➀Natural scenery ➁air quality ➂social leisure	➀Natural scenery ➁air quality ➂social leisure

Adjective words used more	➀Pleasant ➁close ➂vitality	➀Pleasure ➁closeness ➂vitality, freshness	➀Pleasant ➁close ➂vitality	➀Pleasant ➁close ➂vitality	➀Pleasant ➁close ➂vitality	➀Pleasant ➁close ➂vitality

Attracting visual elements	➀Natural elements ➁plant colors and species ➂waterfront design	➀Natural elements ➁plant colors and species ➂waterfront design	➀Natural elements ➁plant colors and species ➂waterfront design	➀Natural elements ➁plant colors and species ➂waterfront design	➀Natural elements, ➁plant colors and species ➂waterfront design	➀Natural elements ➁plant colors and species ➂waterfront design

Characteristic feeling	61.8% can feel the unique beauty	77.8% can feel the unique beauty	75% can feel the unique beauty	66.7% can feel the unique beauty	100% can feel the unique beauty	76.8% can feel the unique beauty

#### Cyclists’ Cognitive Maps

**FIGURE 3:**
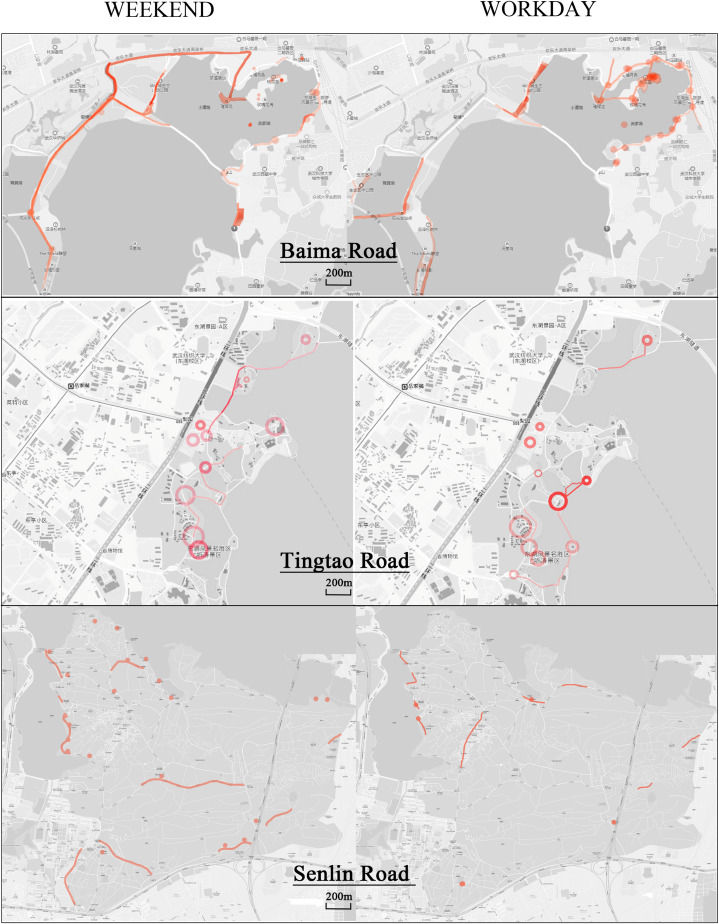
Baima Road, Tingtao Road, Senlin Road Cognitive map. *Color depth indicates the degree of impression of the landscape in the area.

After superimposing the image nodes and paths drawn by the interviewed cyclists, a preliminary imagery space diagram was obtained (Figure 3). Many had difficulties in identifying the physical location of landscape imagery and were only able to provide a fuzzy spatial scope area. Others were quick to identify the impression of the pictured landscape node and were able to provide a detailed description of the landscape characteristics and their feelings related to the scene.

### Features of Landscape Visual Elements

With the help of an App, “Cats’ Eye Quadrant” ⑫, we were able to quantify the number of people, number of motor vehicles, and the area rate in photographs (Table 6). The number of vehicles can reflect on-site activity conditions and the degree of motor vehicle interference. The area rate reflects the visual structure of the landscape. Relationships between node data and equidistant record data can also reflect a given rider’s preferences.

**TABLE 6:** Landscape elements indicators.

Study		Observation	Number of	Number of	Number of motor	Building area	Green	Road area	Sky area
Object	Day	type	photos	people	vehicles	ratio(%)	rate(%)	ratio(%)	ratio(%)
Baima Road	Workday	Isometric Average	86	0.5	0.16	2.97	39.3	29.28	20.93
		Node Average	115	0.12	0	5.97	50.24	9.56	24.11
	Weekend	Isometric Average	85	2.1	0.08	5.2	40.32	20.45	22.54
		Node Average	112	0.63	0.02	5.49	53.68	9.23	19.52
Tingtao Road	Workday	Isometric Average	40	1.98	1.33	9.08	45.11	23.38	5.77
		Node Average	23	1.86	1.88	5.91	48.85	17.77	0
	Weekend	Isometric Average	39	3.97	0.97	7.65	48.85	17.88	8.25
		Node Average	20	4.22	1.43	4.89	57.55	12.25	10.01
Senlin Road	Workday	Isometric Average	181	1.22	0.29	3.33	51.85	20.64	14.76
		Node Average	203	0.49	0.19	5.68	54.21	14.95	15.96
	Weekend	Isometric Average	180	2.17	0.5	3.81	51.14	18.6	15.41
		Node Average	205	1.08	0.21	4.76	55.98	14.25	15.65

From the results of the photo analysis, the average green viewing rate of the three green roads reached 49.76%, the sky rate was 14.4%, the road 17.35%, and the building at about 5.4%. Natural elements occupy a dominant position in the landscape vision, indicating that the overall quality of the landscape on the greenway cycling route is relatively good, but at the same time the lowest green viewing rate was 39.3%. This may be related to the fact that Baima Road is still in the optimization period, and some street trees have only been planted for a short time and their growth is limited. Tingtao Road, which was originally a private garden, is now easily accessible by visitors and is known for its historical and cultural heritage. However, the high frequency of motor vehicles may interfere with slower-moving activities. Additionally, this area did not show significant variation in relation to the time period. Senlin Road is the longest and most complex trail in this study, and also the area with the most abundant landscape. The data show that activities varied considerably among equidistant records and node photographs. Combined with its landscape factor ratio, the overall green viewing rate was high, as expected due to the expansive natural landscape of this route.

### Correlation Analysis of Landscape Perception

#### Score and Rider Attributes

The social characteristics of cyclists indicated that the main beneficiaries of the greenway are still primarily the local population and that preferences with respect to gender, age, and career are evident (Table 7). Male cyclists gave higher scores than women, indicating a potentially higher stress level for women than men that was not resolved during the greenway experience. Overall assessments by elderly and very young users were higher than those of young to middle-aged users. Middle-aged participants, as the main users, wanted more from their landscape environment. The overall low score given by professionals and design-related cyclists indicated their need for a greater range of experiences, longer cycling routes, and the potential incorporation of landscape features that allowed for special cross-regional activities.

**TABLE 7:** Cyclists’ assessment scores and features.

Features Scores	Features	3–5	5–7	8–10	Total
Gender	Male	0	57	136	193
	Female	11	23	45	79
Career	Related to Design	12	34	125	171
	Not Relevant to Design	11	45	45	101
Age	Below 18	0	0	12	12
	18–25	11	45	22	67
	26–30	0	11	23	34
	31–40	0	23	34	67
	41–50	0	0	45	45
	51–60	0	0	12	12
	60+	0	0	34	34

#### Score and Node Spatial Distribution

**FIGURE 4:**
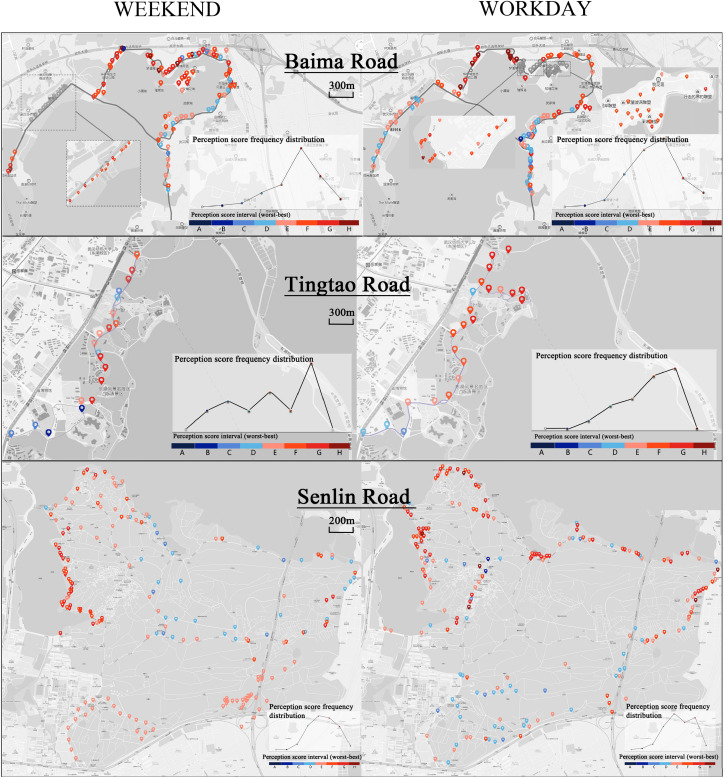
Baima, Tingtao, and Senlin Road weekend and workday node assessment spatial distribution map.

By assigning a score for each node to the space, a node assessment chart was obtained covering the three roads for weekends and workdays (Figure 4). The chart visually reflects the nodes that are more likely to have left a positive visual impression.

Judging from the spatial planning characteristics of the three greenways, they all make full use of the lakeside shoreline, especially Baima and Tingtao. Many landscape nodes are densely distributed in the waterfront area. Setting the cycling route in the waterfront area ensures sufficient visual openness. Therefore, the convex side of Baima has higher average scores than the concave side, and the whole of Tingtao is relatively homogeneous. At the same time, the waterfront area node of Senlin will be higher than the overall level of the mountain area.

In the design of the greenway environment, more public art sculptures are arranged on both sides of the Baima greenway route, which will also greatly attract the attention of cyclists and increase the attractiveness of the area. The Tingtao greenway route is surrounded by urban cultural public buildings (museums, libraries), entertainment venues (ocean parks, playgrounds), and residential buildings, yielding a relatively high vitality index that also increased the overall score. The design of Senlin also has these features. The western waterfront area is not only close to the university campus living area but also has creative bookstores, barbecue camping areas and other places to enhance vitality. In contrast, the east side is bounded by a busy road and the landscape feel and atmosphere of activities are both relatively poor.

Comparing the data on weekdays and weekends, it was found that the nodes in the middle and high scoring segments were appropriately reduced on weekends, and the degree of aggregation of nodes was appropriately increased. From the interview survey, it can be deduced that this is due to the crowding and lack of security caused by the increase in the number of weekend activities.

#### Scores and Visual Elements

**FIGURE 5:**
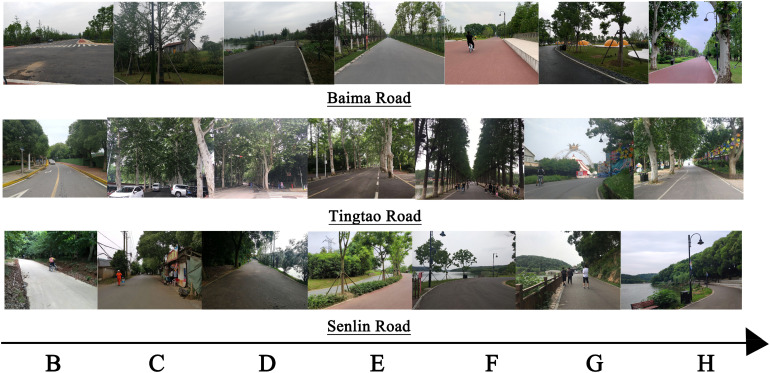
Typical landscape of different segments.

Analysis of the pictures of the typical landscape nodes of the three roads (Figure 5) revealed that the proportions and positions of natural features conformed to the laws of graphical esthetics (Klix, 2001; Wagemans et al., 2012), and the openness and brightness of a space objectively affected the visual perception of cyclists (Zhang et al., 2019). Structures with a sense of design can form stimuli in the scene, prompting cyclists to engage in conditioned reflection and cognitive imagery (Momtaz and Daliri, 2016).

Further statistical analyses of the feature area visitation rate and scoring results (Tables 8, 9) were conducted to determine the internal relationship among factors. A negative correlation was identified between the perception of greenness and the number of cars, whereas building area and number of cars were positively correlated.

**TABLE 8:** Analysis of net correlation of objective factors.

	Number of	Number of motor	Building area	Green	Road area
	people	vehicles	ratio (%)	rate (%)	ratio (%)
**Number of people**					
Relevance	1.000	0.179	0.139	-0.245	0.216
Visibility		0.008	0.040	0.000	0.001
Df	0	216	216	216	216
**Number of motor vehicles**					
Relevance	0.179	1.000	-0.016	-0.133	0.275
Visibility	0.008		0.817	0.050	0.000
Df	216	0	216	216	216
**Building area ratio(%)**					
Relevance	0.139	-0.016	1.000	-0.440	0.024
Visibility	0.040	0.817		0.000	0.725
Df	216	216	0	216	216
**Green rate(%)**					
Relevance	-0.245	-0.133	-0.440	1.000	-0.456
Visibility	0.000	0.050	0.000		0.000
Df	216	216	216	0	216
**Road area ratio(%)**					
Relevance	0.216	0.275	0.024	-0.456	1.000
Visibility	0.001	0.000	0.725	0.000	
Df	216	216	216	216	0

**TABLE 9:** Analysis of objective elements and scoring distance.

	Euclidean					
	
	Number of	Number of motor	Building area	Green	Road area	Sky area
	people	vehicles	ratio (%)	rate (%)	ratio (%)	ratio (%)
Number of people	0.000	61.457	252.911	1382.211	421.621	515.130
Number of motor vehicles	61.457	.000	256.328	1396.189	426.911	527.689
Building area ratio(%)	252.911	256.328	0.000	1332.513	429.879	507.947
Green rate(%)	1382.211	1396.189	1332.513	0.000	1210.790	1147.075
Road area ratio(%)	421.621	426.911	429.879	1210.790	0.000	490.002
Sky area ratio(%)	515.130	527.689	507.947	1147.075	490.002	0.000
Overall rating	378.880	390.379	414.867	1218.705	468.670	475.413

Our statistical results show that the green viewing rate had the greatest influence on the overall perception score. The remaining factors did not differ significantly in their influence, although the influence of roads and sky was high. Thus, for the overall landscape structure, green space and its related feelings had the greatest influence on visual perception (Kaplan and Kaplan, 1989; Maas et al., 2006; Jiang et al., 2014; Lindala and Hartig, 2015).

### Imagery and Perception Elements

**FIGURE 6:**
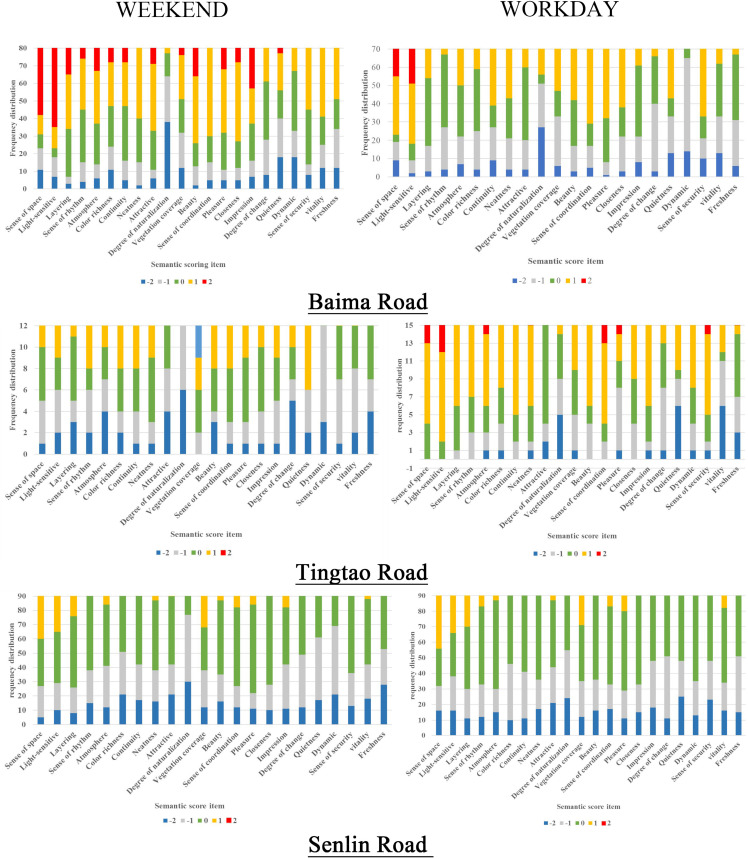
Baima, Tingtao and Senlin Road weekend and workday nodes of Landscape perception levels.

To further study which factors influenced the final perception score, the scores for each node in the three roads were analyzed separately (Figure 6), and the factors influencing different imagery structures were examined.

The Baima Road landscape makes full use of natural resources such as the mountains and water and the core position of the island in the lake. Thus, the entire cycling experience forms a coherent imagery space. As can be seen from the frequency chart, cyclists evaluated their overall sense of space, security, and coordination, followed by impression and beauty, and perceived deficits in the natural quality of the experience. According to the semantic description of typical nodes in each segment, the reasons for low scores were a lack of security (e.g., wide roads and motor vehicles) and disharmony in the landscape (e.g., architecture, municipal facilities). Interesting and novel sculptures, parking facilities, and rest facilities were key elements that appealed to cyclists. In addition to considering the road itself, cyclists also noted whether the surrounding environment showed change and natural rhythms, natural culture with rich connotations, and ecological diversity.

Tingtao Road is a traditional humanistic park-style greenway. Cultural resources are used to enhance the sense of experience and participation. Thus, cyclists can continuously enjoy the cultural scenery as forming a linked imagery space. The assessments for a sense of space, atmosphere, and coordination were high. The degrees of naturalization, quietness, and overall public opinion were more variable, revealing greater differences. Although vegetation cover was well recognized, the assessment of its natural quality was not high, indicating that the greenway landscape maintenance and restoration were not sustained. Combining this information with the analysis of the semantic description of typical nodes, we concluded that cyclists generally believed that plant maintenance management was poor. Some sections of the environment were described as “claustrophobic” and “not secure,” with buildings obscuring the view. Neat and micro-transformed beautification nodes were preferred.

Cyclists in Senlin Road appeared to pay more attention to the route design and natural sense. Leisure activities and artistic sculptures enriched the cyclists’ experience. Similar nodes combined with each other to form a fragmented imagery space. Cyclists gave high scores to the sense of space, followed by vegetation cover and attractiveness, with low scores for naturalization, freshness, dynamics, and security. Thus, the overall landscape design takes full account of the use of natural resources (Benjamin and Roland, 2002); however, certain deficits were evident in the humanized design and subsequent management. Combined with the semantic description, these data show the engineered structures and traffic facilities within the overall landscape were unsatisfying to cyclists. The cyclists preferred dedicated bicycle lanes, an improved route marking system, a wide field of view, and rich plantings/green space. According to the semantic description of the typical node, some sections (e.g., barbecue areas and parking areas) did not allow one to experience the greenway landscape. Thus, further management and consideration of the layout of commercial spaces in the scenic area are required. At the same time, rich vegetation and beautiful water scenery with a sense of designed construction (structures) were met with great enthusiasm by the cyclists.

Combined with the theory of spatial imagery, some impressive landscape node scenarios were in line with the principles of figure-ground, continuity, similarity (Arnheim and Shouyao, 1998), and fragments of broken imagery that ultimately constitute the imagery world of cyclists. Visual information was processed at psychological and esthetic levels (Valerie Gray Hardcastle, 2001). The cultural features of the imagery pattern can be considered in combination with the social characteristics of cyclists for further analysis.

## Discussion

Through the collection of indicators, it was found that cyclists offered positive assessments of the greenway landscape as a complex. In addition to choosing words with relatively positive meanings such as “pleasant, close, and vital,” many cyclists also selected adjectives that focus on unsatisfactory environmental details. Different types of greenway visual landscape, sections, nodes of the same greenway, and even the same node in different time periods obtained different visual perception results, which fully confirms the subjective nature of visual assessment. The objective material environment still exists in a strongly subjective world – a view confirmed in this research (Zhou et al., 2014). However, the landscape imagery tended to produce a memory and cognitive blur due to the lack of effective venue memory for specific nodes along the way (Isola et al., 2011). In random interviews, the differences among the subjective values of cyclists were considerable, as reflected in their judgments of the same scene. Their judgments also tended to be full of emotionally evocative adjectives.

This paper presented innovative research on the correlation between visual perception and landscape imagery for cyclists in the greenway, and some preliminary results were shared. There are limitations to the research design: One is that there is a compound effect between vision and other senses, especially hearing (Mack, 1979; Xuan, 2019). Many respondents mentioned the sound of running water or the sound of birds, and eye movement experiments could be considered as one way of improving the inclusion of this aspect (Ren and Kang, 2015; Xiangyi, 2017). The second is the neglect of the differences in the continuity of cycling routes, just like in many previous studies using photographs (Shen, 2005; Gan et al., 2014; Gebru et al., 2017). The third is that the understanding of visual perception is not deep enough (Tveit, 2009). Fourth, some scholars have proposed that cultural background is also an influencing factor of image perception (Coltheart, 2004; Stobbelaar and Pedroli, 2011).

## Conclusion

### Landscape Imagery Structure

The innate natural conditions and landscape design of different greenways are perceived in unique ways depending on cyclists’ visual perception. The meaning of the space will differ accordingly, leading to differences in the local landscape imagery assessment. The three types of greenways in Phase II form a coherent, connected, and scattered (fragmented) imagery structure in the minds of cyclists.

### Influencing Factors of Assessment

The needs of cyclists are relatively abstract in visual terms. However, research can translate these needs into continuous paths, safe environments, open landscapes, and rich human experiences. Natural elements such as plants and the sky are intrinsically more popular than artificial elements. A greenway with more linear paths for better visualization of the road ahead is the most basic need of cyclists. Additionally, if the field of vision is open, various elements are perceived to be instantaneously more pleasant in contrast to more crowded social areas with people and/or animals. The combination of these elements has an impact on perceptual assessment.

Citations:

➀The Pearl River Delta, located in the south-central part of Guangdong Province, is an important economic center in China.

➁Based on the statistical results of research conducted by CNKI and WOS with the theme of “Greenway cycling.”

➂*Guidelines for Greenway Design and Planning(2016)*,published by Ministry of Housing and Urban-Rural Development of PRC, is to guide scientifically plan and design greenways, to improve the level of greenway construction, and give full play to the comprehensive functions of greenways.

➃The difference between the two esthetic viewpoints is whether the essence of beauty is “appearing beautiful” or “really beautiful.” Kant only regards “sensory perception as the art of yardstick” and believes that true beauty always implies its moral consciousness and moral emotions. There is a fundamental difference in “appearing beauty” that only inspires sensual pleasure.

➄*European Landscape Convention*, published by European Landscape Convention of the Council of Europe,2000. Official website: https://www.coe.int/en/web/landscape/home.

➅According to the *Regulations on Scenic Spots (2006)* of the PRC, national-level scenic areas are approved by the State Council to be announced as national-level scenic areas. It refers to an area with ornamental, cultural, or scientific value, relatively concentrated natural landscapes and cultural landscapes, and a beautiful environment for people to visit or conduct scientific and cultural activities.

➆Eastlake has a long history, so many historical allusions are cited when naming the scenic spots and greenways: Baima refers the war horse of Lu Su, a famous general from the Three Kingdoms period of China in 208 A.D., was buried here; Tingtao scenic area was the private garden of the national capitalist Zhou Cangbai. After transformation, it became a public space, known as “One Scenery and One Garden, Listening to the Waves and Enjoying Pears”; Senlin means forests.

➇Golledge believes that people’s spatial cognition process is three-stage, and a specific “anchor points” plays a role in constructing an information system. Golledge R. Learning about urban environments[A]. In: Carlstein T, Parkes D, Thrift N. Timing space and spacing time, Vol.1[C]. London, Edward Arnold, 1979.

➈The climatic conditions on the survey days are as follows: weekend days, cloudy, 17–26°C, relative humidity 70%, level 4–5 northeast wind; workdays, cloudy, 30°C, relative humidity 59%, southeast wind level 3–4.

➉The cyclists’ standard horizontal viewing angle was about 45°, and the vertical angle of view ranged from 26° to 30°.

⑪According to the statistical report of tourists on Eastlake Greenway, the average daily number of bicycle tourists is about 1375. The sampling rate of this random questionnaire survey is about 5.8%.

⑫“Cat’s Eye Quadrant” is a survey and observation tool launched by the company *Urban Quadrant* based on mobile Internet and computational vision technology. The program can identify the number of people, vehicles, green viewing rate, sky rate and other indicators, where the green viewing rate and sky rate respectively refers to the area of the green plants and blue sky as a proportion of the total area of the photo. Company website: http://www.urbanxyz.com/.

## Data Availability Statement

The datasets generated for this study are available on request to the corresponding author.

## Ethics Statement

Huazhong University of Science & Technology Human Ethics Committee waived the requirement for ethical approval for this study due to the following reasons: the study doesn’t involve involuntary personal information investigations, and it doesn’t involve any violation of the law. Written informed consent was obtained from the minor(s)’ legal guardian/next of kin for the publication of any potentially identifiable images or data included in this article.

## Author Contributions

HH proposed the research direction. JL completed the experimental design and analysis and wrote the article. XL participated in the field research and proposed the revision suggestion. YY participated in the field research and partial data analysis. All authors contributed to the article and approved the submitted version.

## Conflict of Interest

The authors declare that the research was conducted in the absence of any commercial or financial relationships that could be construed as a potential conflict of interest.
